# Fitting in or Not Fitting in: Cultural Congruity as a Correlate of Motivation for Intergroup Contact

**DOI:** 10.3390/bs16060921

**Published:** 2026-06-04

**Authors:** Marina M. Doucerain, Myra Deraîche, Lisa Stora, Paul R. Carr, Alhassane Balde

**Affiliations:** 1 Département de Psychologie, Université du Québec à Montréal, Montreal, QC H3C 3P8, Canada; 2 École des Langues, Université du Québec à Montréal, Montreal, QC H3C 3P8, Canada; 3 Department of Psychology, University of Guelph, Guelph, ON N1G 2W1, Canada; 4 Départment des Sciences de l’Éducation, Université du Québec en Outaouais, Gatineau, QC J8X 3X7, Canada; 5 Département d’Éducation et Formation Spécialisées, Université du Québec à Montréal, Montreal, QC H3C 3P8, Canada

**Keywords:** intergroup contact, intercultural twinnings, motivation, cultural congruity

## Abstract

It is well established in social psychology that intergroup contact is beneficial to reduce intergroup bias. Based on this insight, a growing body of work has focused on correlates of people’s motivation to seek or avoid intergroup contact. The present study contributes to this literature by probing motivation for an ecologically valid and ideal form of intergroup contact: intercultural twinnings (structured exchange activities between people of diverse linguistic and ethnocultural backgrounds). We examined three facets of motivation (contact willingness; intrinsic motivation; and contact opt-in as a proxy for behavioral intent) and a range of well-established intergroup contact associates. We also tested the role of cultural congruity with the dominant society, inspired by push–pull theories of migration. Participants included 214 students in Québec, Canada. The results show that motivational profiles differed depending on motivation facets. Intergroup anxiety was negatively related to intrinsic motivation; desire for self-expansion was positively related to all three facets; ethnocentrism was negatively associated with our behavioral proxy. Cultural congruity was associated with all three facets through a suppression effect, such that greater perception of not fitting in Québec society was related to higher motivation indices once avoidance dispositions were taken into account. With prevalent intergroup tensions, better understanding how to “bring the horse to the contact water” is essential.

## 1. Introduction

The notion that intergroup contact reduces intergroup biases has become received knowledge in social psychology (Pettigrew & Tropp, 2006). Yet, daily interactions across an ethnocultural divide are scarce. As a case in point, in a daily diary study, participants within the ethnocultural majority reported no substantive intercultural interaction at all in more than a quarter of daily diaries (Doucerain et al., 2023). Faced with this conundrum, an increasing number of studies have investigated what motivates people to seek or avoid intergroup contact, typically focusing on how much motivation people have at the expense of motivation type. In the present work, we contribute to this emerging body of work by probing motivation for a locally relevant form of intergroup contact: intercultural twinnings (described below). We examine three facets of motivation—contact willingness, intrinsic motivation, and contact opt-in—as a proxy for behavioral intent. We also test the role of cultural congruity with the dominant society—perceptions of how well one fits in—a novel correlate inspired by push–pull theories of migration.

### 1.1. Intercultural Twinnings

Intercultural twinnings are structured exchange activities between people of diverse linguistic and ethnocultural backgrounds, with the goal of learning from each other and developing intercultural and communicational skills along the way (Carignan et al., 2022). More broadly, they seek to improve social harmony in multicultural settings. They can take place in a variety of contexts, such as community organizations or educational institutions. In our local university context, intercultural twinnings—translated from the French “jumelages interculturels”—typically involve the pairing of students who have recently arrived in Québec and are learning French with majority students (i.e., born or raised in Québec with French as their dominant language). Since the early 2000s, an estimated 15,000 students have taken part in intercultural twinnings at our university, in a variety of departments, including French as a second language, psychology, social work, or sociology. These exchange activities are organized by interested and willing instructors/professors and are structured as part of course requirements. They can vary in focus and modalities (e.g., number, length, and topic of meetings), but they all share a common goal of building cultural bridges.

To illustrate, a professor teaching French as a second language may decide to pair up with a professor teaching psychology for one semester. Together, they may decide that their students will meet four times during the semester to have unstructured conversations lasting about one hour each on a given list of topics. These conversations would be mandatory for students in both courses and provide the basis for graded coursework. Students in the French as a second language course may have to create topical vocabulary lists based on their discussions, whereas students in the psychology course may have to write an essay on the intergroup concepts and theories that manifested during the discussions. The instructors may or may not decide to renew the experience the following semester and the students may or may not encounter intercultural twinnings in other courses.

In many ways, intercultural twinnings are designed to approximate an optimal form of intergroup contact, as they intentionally incorporate key enabling conditions identified by Allport (1954). First, equal-status interaction is structurally supported, as both participants are students enrolled in parallel courses and engage as peers in a shared academic activity. Second, shared goals and cooperation are embedded in the design of the program: interactions are tied to course assignments that require mutual participation (e.g., discussions forming the basis for graded work), thereby creating interdependence. Third, institutional support is explicit, as twinnings are organized and endorsed by instructors and integrated into course requirements, signaling normative approval from relevant authorities. In many cases, instructors also incorporate elements of guided reflection (e.g., written assignments connecting experience to course content), which may further support perspective-taking. Finally, intercultural twinnings may create opportunities for relationships to extend beyond the structured activity, aligning with later extensions of contact theory emphasizing the role of friendship (Pettigrew, 1998). These structural features may also shape participants’ expectations and motivation to engage, insofar as institutional endorsement and clearly defined interaction norms can reduce uncertainty and legitimize contact.

At the same time, the specific implementation of these features can vary across courses (e.g., degree of structure, presence of guided reflection), suggesting that intercultural twinnings should be understood as a flexible program that generally instantiates these conditions rather than uniformly guaranteeing them. Past research has elucidated that intercultural twinnings can be effective in reducing intergroup bias, such as stereotypes and prejudice (Carignan et al., 2014, 2022).

Intercultural twinnings are also useful for studying intergroup contact motivation. Since they typically require significant time investment from participants, one’s expression of motivation to partake in twinnings is likely less prone to social desirability than generic questions such as “Would you be willing to interact with a member of social group X?” This is even more the case given that participants have to participate in them as part of course requirements. Further, Paolini et al. (2022) have pointed out that research on drivers of intergroup contact motivation is typically acontextual. It tends to rely on very broad measures of willingness to have contact with the outgroup in general, or with unspecified outgroup members. These authors underscore the need for ecologically valid and situated contact motivation studies. Intercultural twinnings are well-established intergroup contact activities in our local context—they are, for example, the core topic of a university-funded research group. As such, they are meaningful and relevant to our participants. For these reasons, we focus here specifically on the motivation to take part in intercultural twinnings.

### 1.2. Intergroup Contact Motivation

A growing body of work has investigated people’s motivation to engage in intergroup contact, using a variety of conceptualizations and terms, such as contact *seeking* (Paolini et al., 2018), *willingness* (Ron et al., 2017), *interest* (Migacheva & Tropp, 2013), *motivation* (Halperin, 2012), *volition* (Bagci et al., 2021), or *intention* (Wang et al., 2020). This heterogeneity bespeaks clear common underpinnings, but it also reflects important nuances in how we understand people’s drive and interest to engage in intergroup contact. It may also limit our ability to derive actionable insights from research results. For example, correlates of one’s willingness to interact with an outgroup member might differ from one’s intention to do so, something we target here. Further, this research has focused on factors associated with *how much* willingness or motivation people have, largely ignoring *what kind* of motivation is at play. Research on what shapes the quality or orientation of intergroup contact motivation is scarce (Böttcher & Friehs, 2022). With these points in mind, we consider three facets of intergroup contact motivation in the present work. We measure the traditional contact willingness, typically measured with items such as “I would be happy if I had an opportunity to engage in contact with [outgroup member] (Voca et al., 2023) or “How interested would you be in striking up a conversation with [outgroup member]?” (Wang et al., 2020). We also measure a proxy of behavioral intention to take part in intercultural twinnings and intrinsic motivation—a novel feature of this work. We explore whether documented antecedents of intergroup contact motivation are differentially associated with these three facets of contact motivation.

#### Intrinsic Motivation

As a comprehensive theory of human motivation, self-determination theory articulates that people vary not only in their motivation level (i.e., low or high motivation) but also in the type of their motivation. At the most basic level, this theory distinguishes between intrinsic and extrinsic motivation as two main types of motivation based on different reasons or goals to pursue an activity (Ryan & Deci, 2000). Intrinsic motivation refers to doing an activity as its own end because it is considered rewarding in itself. For example, someone taking part in an intercultural twinning because they find it enjoyable and enriching would be intrinsically motivated. In contrast, extrinsic motivation refers to doing something for an instrumental goal or to obtain external rewards. For instance, taking part in an intercultural twinning to be seen as open-minded by friends would reflect extrinsic motivation. Self-determination theory proposes that intrinsically motivated behavior bolsters well-being (Ryan & Deci, 2000). In the context of intergroup relations, intrinsic motivation may be more effective in reducing prejudice (Böttcher & Friehs, 2022; Legault & Amiot, 2014), which is why we focus on this motivation type here. In addition, the mandatory nature of intercultural twinnings in our local context is likely to flatten variations in extrinsic motivation among participants.

### 1.3. Correlates of Intergroup Contact Motivation

Research on correlates of intergroup contact motivation is proliferating. Three recent reviews (Kauff et al., 2021; Paolini et al., 2018; Ron et al., 2017) have identified a wide range of drivers, grouping them in micro-, meso-, and macro-level influences (with some nuances in how these levels are construed across reviews). Macro-level drivers reflect the broader societal context, such as the influence of cultural norms or institutional characteristics. For example, the communication of ethnic tolerance norms on a daily radio program was reported to have had improved intergroup behaviors between the Tutsi and Hutu people in Rwanda (Paluck, 2009). Meso-level drivers relate to intragroup and intergroup processes, such as group status or history. For example, on average, members of dominant (e.g., White Francophones in Quebec) versus minority (e.g., Asian immigrants in Quebec) groups seek different types of intergroup contact and have different motivations to do so. The former generally prefer avoiding topics of power difference (Saguy & Kteily, 2014) and can sometimes see intergroup contact as a way to distance themselves from past ingroup wrongdoing (Mazziotta et al., 2014). In contrast, the latter often prefer to address power differences and inequality with the goal of improving their ingroup’s position (Ron et al., 2017; Saguy & Kteily, 2014). Having a history of more extensive intergroup contact is also positively associated with contact motivation (Kauff et al., 2021).

Finally, micro-level correlates pertain to individuals’ dispositions and preferences, such as prejudice or personality. For example, a longitudinal field survey in Germany, Belgium, and England with ethnic minority and majority school students showed that participants with more negative attitudes initially were more likely to avoid outgroup contact, even when opportunities were available (Binder et al., 2009). In terms of personality traits, greater openness and extraversion are positively linked to intentions to interact with individuals within ethnocultural outgroups (Stürmer & Benbow, 2017), whereas associations are negative for social dominance orientation and right-wing authoritarianism (Rosenthal & Levy, 2012). There is also strong longitudinal and experimental evidence that people with high intergroup anxiety actively avoid intergroup contact in general (Paolini et al., 2018). Lastly, recent research has identified a range of appetitive motivations (seeking something positive rather than avoiding something negative) prompting people to seek intergroup encounters (Kauff et al., 2021). Some reflect pragmatic or social goals, such as professional advancement or group-image concerns (Stürmer & Benbow, 2017), but others reflect a desire for personal growth or self-expansion. Self-expansion stems from a core human motivation to expand the self to include “otherness,” in the shape of material or social resources, identities, or meanings (Wright et al., 2003). Paolini et al. (2016) found that this appetite for “different” others was associated positively with interest in intergroup interactions. In short, micro-level factors documented thus far fall into two rough categories: negative appraisals of the outgroup (e.g., prejudice, anxiety) driving one to avoid it versus appetitive inclinations prompting one to seek the outgroup (e.g., self-expansion). The various correlates mentioned above are summarized in Table 1. Missing from this body of work is an examination of factors pushing one to seek the outgroup because of discomfort in one’s own social environment. We consider such a push factor in the present study as one additional correlate of motivation for intercultural twinnings.

### 1.4. Push Factors and Motivation for Intercultural Twinnings

Lee’s (1966) theory of migration posits that migrants’ motivation and decision to migrate stem from pull and push factors. The former refer to positive characteristics of the new area attracting people there, such as good employment opportunities or desirable living conditions. In contrast, the latter refers to negative characteristics of the place of origin compelling people to leave it, such as an oppressive regime or discrimination. Conceptually, immigration shares some features with intergroup contact, both manifesting a “move” to a different social space. As such, a push/pull framework may be helpful in making sense of factors shaping intergroup contact motivation. Indeed, the appetitive factors described above, such as self-expansion, align well with migration pull factors. For example, with self-expansion, promising resources, identities or meanings in the outgroup pull people to “move” to this social space through intergroup contact. Push factors have received less attention. Here, we investigate the lack of cultural congruity as one such factor.

#### Lack of Cultural Congruity in Quebec

Cultural fit refers to the consonance between a person’s cultural ways and those normative and valued in their local cultural context (Ward & Chang, 1997). Across domains and constructs—be it in terms of values, self-construal, goals, or emotions—research suggests that greater cultural fit is positively associated with adaptive outcomes (Dressler et al., 2018; Elfenbein & O’Reilly, 2007; Fulmer et al., 2010; Stephens et al., 2012; Yoo & Miyamoto, 2018). For example, taking into account age, gender, and socioeconomic status, Brazilian participants whose views on “having a good life” aligned with the culturally dominant model reported less psychological distress (Dressler et al., 2018). Especially relevant to our present investigation, a meta-analysis showed that lower cultural fit in the organizational context (e.g., person-organization fit) was associated with workers’ greater intention to quit their job (Kristof-Brown et al., 2005). Similarly, some work suggests that first-generation college students may be more likely to drop out than continuing-generation students (i.e., when at least one parent went to university) because of a cultural mismatch between their cultural ways and those of higher education institutions (Devi, 2020). These findings echo work on social capital in sociology (Bourdieu, 1985). In both cases, cultural misfit with their local setting prompts people to move out of it. We propose that something analogous may be at play for intergroup contact. People who perceive a lack of cultural fit in their ethnocultural environment may be more motivated to seek contact in a cultural outgroup than those who feel they fit in well.

Studies of cultural fit sometimes consider objective fit (actual consonance between people’s characteristics and their environment’s) and sometimes perceived fit. Here, we focus on the latter, also referred to as cultural congruity (Gloria & Kurpius, 1996) and defined as people’s subjective perception that one “fits in” in their local ethnocultural context (i.e., Québec). We do so for two reasons. First, cultural congruity is more proximal to attitudes and behavior than objective fit (Cable & DeRue, 2002). It is also more strongly related to adaptive outcomes, at least in organizational (Kristof-Brown et al., 2005; Ravlin & Ritchie, 2006) and higher education domains (Wessel et al., 2008). Second, cultural congruity lends itself well to measurement by self-report questionnaires. As such, it is more practically feasible to measure it in a wide range of contexts and populations, thus making it more broadly accessible to intergroup contact researchers.

### 1.5. The Present Study

Paolini et al. (2022) ran an ‘educational hijab stall’ at two university campuses in Australia, investigating non-Muslim women’s motivation and choice to have contact with Muslim women. With this research, they underscored the need for ecologically valid field research documenting why people naturally seek intergroup contact. In that spirit, we consider correlates of motivation for an optimal form of contact highly relevant in our local university context, intercultural twinnings. Importantly, we distinguish between willingness and intrinsic motivation—a specific type of motivation—and also consider contact opt-in as a proxy for behavioral intent. We investigate a range of documented meso- and micro-level correlates of motivation for intercultural twinnings, exploring their differential associations with these three facets of contact motivation. We also test the role of cultural congruity, a novel potential correlate, inspired by push–pull theories of migration. We hypothesize that lower cultural congruity in Québec will be associated with greater motivation for intercultural twinnings (willingness, intrinsic motivation, and contact opt-in).

## 2. Materials and Methods

### 2.1. Participants and Procedure

Participants were students at two French-speaking universities belonging to the same university network in Québec, Canada. The initial sample comprised 247 students. Among those, 33 did not complete any questionnaires, providing only demographic information. These participants were not retained, leaving a final sample of 214 participants, ranging in age from 19 to 50 (M = 27.10, SD = 7.44). Most students were undergraduate (*n* = 149) and attended the university located in Québec’s largest city (*n* = 177). Thirty-six students attended the university located in a mid-size town (one person did not answer that question). One hundred and fifty self-identified as female, 54 as male, and 9 indicated other gender identities (one person did not answer this question). In terms of ethnicity, 132 participants self-identified as White, 25 as Black, 15 as Arab, 14 as Latin American, 8 as East Asian, 5 as Southeast Asian, 3 as South Asian, 2 as West Asian, and 5 as other (3 persons did not answer this question). Most participants were born in Canada (*n* = 145). Those born outside of Canada (*n* = 69) came from 22 different countries, with France referenced most often (*n* = 11). One hundred and sixty-seven participants had French as their first language (*n* = 167), eight English, with the rest of the sample reporting a variety of other languages.

Participants were recruited in 2021 via flyers placed on both university campuses. Interested individuals were invited to scan the QR code on the flyer, which directed them to the online study hosted on Qualtrics. Participants provided informed consent before answering questionnaires online, which required approximately 40 min of their time. The research protocol was approved by the research ethics boards of both universities (Protocol number 4538_e_2020). Participants received $10 as compensation for their time.

### 2.2. Materials

All study materials were in French. Where necessary, measures were translated from English to French using a committee approach to translation (Douglas & Craig, 2007), which ensures that most time and attention are devoted to problematic content. In this approach, two persons translate the material in parallel and a committee reviews the translations and consensually resolves areas of disagreement. Sociodemographic variables used as covariates included gender identity (male, female, other identity), age, participation in past intercultural twinnings (never vs. at least once), and group status (dominant vs. minority). Before answering contact motivation questions, participants read a brief definition of intercultural twinnings as an organized and supervised activity involving interactions between people of different languages and ethno-cultural backgrounds, in order to learn from each other and build bridges between people of different backgrounds. All internal consistency statistics (McDonald’s ω) were computed on this sample’s data.

#### 2.2.1. Group Status: Minority vs. Dominant

Group status was operationalized using a composite indicator. Participants were categorized as belonging to the dominant group if they were born in Canada, self-identified as White, and reported French as their first language. All other participants were categorized as belonging to a non-dominant (minority) group. This operationalization captures a structural position relative to the francophone White majority in Québec, while necessarily aggregating individuals with diverse ethnocultural, migratory, and linguistic backgrounds. Table 2 provides detailed information on sample composition across the three dimensions used in this operationalization. This approach aligns with intersectionality-informed work conceptualizing group status as a multidimensional structural position rather than a single demographic characteristic (Benkirane & Doucerain, 2022).

#### 2.2.2. Willingness to Participate in Intercultural Twinning (ContWill)

Participants rated their level of agreement or disagreement with 3 in-house statements: “I would like to take part in an intercultural twinning activity”, “I would gladly take part in an intercultural twinning activity”, and “I am motivated to take part in an intercultural twinning activity.” Items were rated on a 7-point scale ranging from 1 *Strongly disagree* to 7 *Strongly agree*. Higher total scores (mean) indicate a greater motivation to participate in intercultural twinnings (ω = 0.96).

#### 2.2.3. Intercultural Twinning Intrinsic Motivation (IntMot)

Participants completed the Intrinsic Motivation Inventory (Deci et al., 1994). In reference to intercultural twinnings, participants rated their level of agreement or disagreement with 8 items such as “This activity would be fun to do” on a 7-point Likert-type scale ranging from 1 *Strongly disagree* to 7 *Strongly agree*. Higher total scores (mean) indicate greater intrinsic motivation for intercultural twinnings (ω = 0.75).

#### 2.2.4. Contact Opt-In (BehProx)

Participants indicated whether they agreed to be contacted by email to take part in future twinning activities. This dichotomous measure (yes vs. no) was used as an indicator of initial commitment (contact opt-in) toward intercultural twinnings. While this measure captures a concrete behavioral step beyond self-reported motivation, it represents a low-cost form of engagement and should not be equated with enacted participation.

#### 2.2.5. Personality Traits

The short version of the Big Five Inventory (BFI; Lang et al., 2011) is a 15-item self-report questionnaire composed of 5 subscales. Based on past studies showing that only extraversion and openness are associated with contact motivation, we included only these two subscales: openness (BFI-O; e.g., “has an active imagination”) and extraversion (BFI-N; e.g., “is outgoing, sociable”). Participants indicated the extent to which each item applies to them on a 7-point scale ranging from 1 *Strongly disagree* to 7 *Strongly agree*. Higher total scores (mean) on each of the 2 subscales indicate higher levels of the corresponding personality trait (ω = 0.78 for openness and 0.76 for extraversion).

#### 2.2.6. Appetite for Cross-Cultural Engagement

The Attitudinal and Behavioral Openness Scale (ABOS; Caligiuri et al., 2000) served as a proxy for cultural self-expansion—people’s appetite for engaging dissimilar others as a source of personal enrichment (Paolini et al., 2016). We used items from two ABOS subscales: Attitudes (ABOS-A), which assesses favorable evaluations of intercultural experiences, and Participation (ABOS-P), which assesses actual engagement in such experiences. Items were rated on a 5-point scale ranging from 1 *Strongly disagree* to 5 *Strongly agree*. A sample item for ABOS-A is “A year-long overseas assignment would be a fantastic opportunity for me and/or my family”. A sample item for ABOS-P is “I attend foreign films”.

Preliminary exploratory factor analyses supported treating these two components separately (see the Supplementary Materials). Accordingly, the Attitudes and Participation subscales were entered as distinct variables in all analyses. Higher scores indicate a stronger appetite for culturally diverse experiences (ω = 0.78 for ABOS-A and 0.79 for ABOS-P).

#### 2.2.7. Outgroup Prejudice

Participants rated their attitudes toward six ethnocultural groups in Québec on a feeling thermometer (Converse et al., 1980) running from 0 to 100 degrees, where lower values reflect “colder” or more negative attitudes and higher values reflect “warmer” or more favorable attitudes. The groups evaluated were White Francophone Quebecers (i.e., attitudes toward Quebec’s dominant group), White Anglophone Quebecers, Quebecers from cultural minorities, Quebecers from racialized groups, Quebecers from religious minorities, and people who immigrated to Québec. Mean scores for the six latter groups yielded a measure of attitudes toward Québec’s minorities (Therm-Min; ω = 0.88).

Our outgroup prejudice variable was tailored to participants’ group membership (i.e., assessing attitudes toward the relevant outgroup for each respondent). As such, it captures a within-person disposition toward the salient outgroup, rather than a strictly comparable attitude toward a single target group across participants. For participants in the dominant status group, intergroup prejudice was operationalized as their attitude score toward Québec’s minorities. For participants in the minority status group, intergroup prejudice was operationalized as their attitude score toward White Francophone Quebecers. Scores were reversed such that higher values represent greater prejudice toward the outgroup.

#### 2.2.8. Ethnocentrism

The Revised Ethnocentrism Scale (RES; Neuliep & McCroskey, 2013) is a 22-item (15 are scored, the rest are fillers) questionnaire designed to measure levels of ethnocentrism. Participants rated their degree of agreement or disagreement with items such as “Most other cultures are backward compared to my culture.” Items were rated on a 5-point scale, ranging from 1 *Strongly disagree* to 5 *Strongly agree*. Higher total scores (sum) indicate higher levels of ethnocentrism (ω = 0.92).

#### 2.2.9. Intercultural Contact Anxiety

The Personal Report of Intercultural Communication Apprehension (PRICA; Neuliep & McCroskey, 1997) is a 16-item questionnaire designed to measure the degree of anxiety associated with the idea of engaging in intercultural contact. A sample item is “engaging in a group discussion with people from different cultures makes me tense and nervous.” Items are rated on a 5-point scale, ranging from 1 *Strongly disagree* to 5 *Strongly agree*. Higher total scores (mean) on the scale indicate higher levels of intercultural contact anxiety (ω = 0.91).

#### 2.2.10. Cultural Congruity in Quebec

The Cultural Congruity Scale (CCS; Gloria & Kurpius, 1996) was adapted to the present context (replacing mentions of students and university by Quebecers and Québec) to measure the extent to which participants perceive that they fit in well in the Québec society. Items such as “when I interact with Quebecers, I feel that we are on the same wavelength” were rated on a 7-point scale ranging from 1 *Not at all* to 7 *Completely*. Higher total scores (mean) reflect a greater sense of “fitting in” well in Québec (ω = 0.78).

To provide additional evidence regarding the adapted Cultural Congruity Scale, we conducted exploratory and confirmatory factor analyses. Parallel analysis and Velicer’s MAP criterion both supported a two-factor solution. An exploratory factor analysis revealed a clear and interpretable two-factor structure. A subsequent confirmatory factor analysis demonstrated good model fit, χ^2^(13) = 22.04, *p* = 0.06, CFI = 0.98, TLI = 0.98, RMSEA = 0.06, SRMR = 0.04.

Measurement invariance analyses across dominant and minority participants supported configural and metric invariance, with minimal changes in fit indices across increasingly constrained models (ΔCFI = −0.005; ΔRMSEA = 0.006). These findings, reported in the Supplementary Materials, suggest that the adapted scale captures two related facets of perceived fit in Québec society that operate comparably across groups.

Notably, the two-factor solution aligned exactly with item wording: one factor comprised all positively worded items, whereas the second factor comprised all negatively worded items that were reverse-scored in the main analyses. As a result, the observed structure may reflect not only substantively distinct facets of perceived cultural fit, but also a method effect associated with item wording. Given this possibility, the substantial correlation between the two factors (r = 0.48) and the theoretical focus on overall perceived cultural congruity, the analyses reported herein retained the composite score including all items. We report exploratory analyses splitting CCS into two factors in the Supplementary Materials.

### 2.3. Analysis

The data that support the findings of this study, along with the analysis script and the study materials, are openly available in the Open Science Framework at https://osf.io/yux3t/overview?view_only=eb69c07965ee426ba09f5ecc84dd42a5, accessed 23 May 2026. Concerning data preparation, we proceeded as follows. First, within each questionnaire, missing item responses were imputed with expectation maximization before total score computation. This approach decreases bias and increases power compared to computing prorated scale scores by averaging the available items (Gottschall et al., 2012; Mazza et al., 2015). Second, for numerical variables (total scores and age), univariate outliers were “winsorized”, whereby values outside of three standard deviations around the mean were brought to the limit of that interval. Three values were modified (0.17% of values): one for age, one for intercultural anxiety and one for outgroup prejudice. Third, missing data on study variables (e.g., total scores) were few (4.58%) and missing completely at random (MCAR), based on the results of a non-parametric test of homogeneity of covariance, *p* = 0.32 (Jamshidian & Jalal, 2010). They were imputed using multiple imputations (with 30 imputations; Graham et al., 2007), using the R package Amelia (Honaker et al., 2011). Regression results were pooled with the Barnard–Rubin adjusted degrees of freedom method (Barnard & Rubin, 1999), using the R package miceadds (Van Buuren & Groothuis-Oudshoorn, 2011).

The hypotheses were tested using multiple regressions. A sample size of 198 would provide sufficient statistical power (set at 0.80) to detect a small to medium effect size of β = 0.20 (estimated f2 = 0.04) with linear regression with 10 covariates (WebPower; Zhang & Yuan, 2018). With N = 214, power was deemed sufficient for this study. We ran three sets of multiple regressions with contact willingness, intrinsic motivation and behavioral proxy as dependent variables, using R (R Core Team, 2024). Independent variables were entered hierarchically with sociodemographic covariates in a first step (age, gender identity, group status, history of past twinning), established correlates of intergroup contact seeking in a second step (personality traits, appetite for cross-cultural engagement, ethnocentrism, intergroup interaction anxiety, intergroup prejudice), and cultural fit in Québec in a third step. All regression models were specified a priori based on theoretical considerations. Linear regression statistical assumptions of residual normality, linearity, and homoscedasticity were verified and found unproblematic (please see the Supplementary Materials).

To examine the discriminant validity of willingness to participate and intrinsic motivation, we conducted exploratory factor analyses using all items from both measures. Parallel analysis supported a two-factor solution, but Velicer’s MAP criterion favored a single-factor solution. A confirmatory factor analysis confirmed that a two-factor solution was a better fit than a one-factor solution, χ^2^(1) = 117, *p* < 0.001, ΔCFI = −0.08; ΔRMSEA = 0.04. These results suggest that willingness and intrinsic motivation are closely related—both reflecting a general motivational core—but that they are still distinct facets of motivation for intercultural twinnings.

## 3. Results

### 3.1. Descriptive Results

Table 3 displays descriptive statistics and correlations among numeric variables. Overall, participants reported moderately high levels of contact willingness (ContWill) and intrinsic motivation (IntMot) to take part in intercultural twinnings. The majority of the sample (64%) agreed to be contacted by email for potential future twinning activities (BehProx). Attitudes toward Québec dominant and minority groups were quite positive (Therm-Maj and Therm-Min, respectively), although attitudes toward Québec’s ethnocultural majority were significantly more positive for participants with dominant status than for those with minority status, *t*(150) = 2.9, *p* = 0.004. On average, levels of ethnocentrism (RES), intercultural interaction anxiety (PRICA), and outgroup prejudice (OutPrej) were fairly low. Appetite for cross-cultural engagement (ABOS) was moderate. Participants’ perception of fitting in well in Québec society (CCS) was moderately high on average, but significantly lower among minority participants than among dominant status participants, *t*(176) = 5.5, *p* < 0.001.

### 3.2. Willingness to Participate in Intercultural Twinnings (ContWill) as Dependent Variable

Table 4 shows the unstandardized results of multiple regressions predicting willingness scores. Sociodemographic covariates introduced in Model 1 significantly improved model fit, R^2^ = 0.10, *F*(5, 205.9) = 4.32, *p* < 0.001. Age was significantly associated with willingness scores, with a medium effect size, β = 0.28, such that older participants reported a greater willingness to take part in intercultural twinnings. Compared to participants who self-identified as female, those self-identifying as male had lower willingness scores, β = −0.33. In Model 2, correlates documented in previous studies were a significant improvement in model fit, R^2^ = 0.28, ΔR^2^ = 0.18, *F*(7, 197.5) = 6.88, *p* < 0.001. Only the ABOS-A scores were significantly associated with willingness scores, with a medium to large effect size, β = 0.41. Thus, participants who reported a greater appetite for cross-cultural engagement also reported a higher willingness level. In Model 3, CCS scores were significantly associated with ContWill scores, with a medium effect size, β = −0.27, R^2^ = 0.33, ΔR^2^ = 0.05, *F*(1, 188.9) = 11.47, *p* < 0.001. Participants who perceived that they fit in less well in Québec society expressed a higher willingness to take part in intercultural twinnings, supporting our hypothesis.

### 3.3. Intrinsic Motivation for Intercultural Twinnings (IntMot) as Dependent Variable

Table 5 shows the unstandardized results of multiple regressions predicting motivation scores. Sociodemographic covariates introduced in Model 1 significantly improved model fit, R^2^ = 0.12, *F*(5, 206) = 5.55, *p* < 0.001. Age was significantly associated with motivation scores, with a medium effect size, β = 0.26, such that older participants reported greater intrinsic motivation for intercultural twinnings. Reporting a female gender identity was associated with higher motivation scores, compared to a male identity, β = 0.34, or a gender identity other than male or female, β = 0.93. In Model 2, correlates documented in previous studies were a significant improvement in model fit, R^2^ = 0.40, ΔR^2^ = 0.28, *F*(7, 197.9) = 12.24, *p* < 0.001. As for willingness, ABOS-A scores were significantly associated with motivation scores, with a medium effect size, β = 0.26. Thus, participants who reported a greater appetite for cross-cultural engagement also reported greater intrinsic motivation for intercultural twinning. The coefficient for PRICA scores was also statistically significant, with a medium effect size, β = −0.34. This indicates that participants reporting greater intercultural communication anxiety were less intrinsically motivated to take part in intercultural twinnings. Greater extraversion, indexed by higher BFI-E scores, was associated with greater intrinsic motivation, with a small effect size, β = 0.13. In Model 3, CCS scores were significantly associated with motivation scores, with a small effect size, β = −0.15, R^2^ = 0.41, ΔR^2^ = 0.02, *F*(1, 192.7) = 4.30, *p* = 0.04. Participants who perceived that they fit in less well in Québec society reported greater intrinsic motivation for intercultural twinnings, supporting our hypothesis.

**Table 4:** Multiple regressions predicting willingness to participate in intercultural twinnings (ContWill).

	Model 1				Model 2				Model 3			
Predictor	*b*	SE	*p*	95% CI	*b*	SE	*p*	95% CI	*b*	SE	*p*	95% CI
Constant	3.56	0.46	<0.001 ***	[2.66, 4.46]	−0.06	1.03	0.957	[−2.09, 1.98]	3.05	1.36	0.026 *	[0.36, 5.73]
Age	0.07	0.02	<0.001 ***	[0.03, 0.10]	0.08	0.02	<0.001 ***	[0.05, 0.12]	0.08	0.02	<0.001 ***	[0.04, 0.11]
Gender identity (male vs. female)	−0.58	0.28	0.036 *	[−1.13, −0.04]	−0.36	0.26	0.173	[−0.88, 0.16]	−0.43	0.26	0.098	[−0.94, 0.08]
Gender identity (other vs. female)	−0.51	0.58	0.383	[−1.65, 0.64]	0.17	0.55	0.756	[−0.92, 1.26]	0.14	0.54	0.792	[−0.92, 1.21]
Group status (minority vs. dominant)	−0.28	0.25	0.265	[−0.77, 0.21]	−0.17	0.23	0.480	[−0.63, 0.30]	−0.39	0.24	0.099	[−0.86, 0.07]
History of past twinning (yes vs. no)	−0.27	0.26	0.301	[−0.79, 0.25]	−0.34	0.24	0.165	[−0.82, 0.14]	−0.35	0.24	0.139	[−0.82, 0.12]
Extraversion (BFI-E)					0.09	0.08	0.290	[−0.08, 0.26]	0.12	0.08	0.157	[−0.04, 0.28]
Openness (BFI-O)					0.01	0.09	0.900	[−0.17, 0.20]	−0.03	0.09	0.764	[−0.20, 0.15]
Appetite for cross-cultural engagement-Attitude subscale (ABOS-A)					0.81	0.14	<0.001 ***	[0.54, 1.09]	0.78	0.14	<0.001 ***	[0.50, 1.05]
Appetite for cross-cultural engagement-Participation subscale (ABOS-A)					0.02	0.16	0.881	[−0.29, 0.34]	0.07	0.16	0.638	[−0.23, 0.38]
Ethnocentrism (RES)					−0.00	0.01	0.905	[−0.03, 0.03]	−0.01	0.01	0.313	[−0.04, 0.01]
Intercultural communication anxiety (PRICA)					−0.23	0.21	0.270	[−0.64, 0.18]	−0.29	0.20	0.155	[−0.69, 0.11]
Outgroup prejudice (OutPrej)					0.00	0.01	0.507	[−0.01, 0.02]	−0.00	0.01	0.743	[−0.01, 0.01]
Cultural congruity in Québec (CCS)									−0.40	0.12	0.001 **	[−0.63, −0.17]

Note: * *p* < 0.05; ** *p* < 0.01; *** *p* < 0.001.

**Table 5:** Multiple regressions predicting intercultural twinning intrinsic motivation (IntMot).

	Model 1				Model 2				Model 3			
Predictor	*b*	SE	*p*	95% CI	*b*	SE	*p*	95% CI	*b*	SE	*p*	95% CI
Intercept	4.35	0.24	<0.001 ***	[3.87, 4.84]	3.26	0.52	<0.001 ***	[2.23, 4.28]	4.22	0.69	<0.001 ***	[2.85, 5.59]
Age	0.03	0.01	<0.001 ***	[0.01, 0.05]	0.03	0.01	<0.001 ***	[0.02, 0.05]	0.03	0.01	<0.001 ***	[0.01, 0.05]
Gender identity (male vs. female)	−0.33	0.15	0.030 *	[−0.62, −0.03]	−0.16	0.13	0.228	[−0.42, 0.10]	−0.18	0.13	0.169	[−0.44, 0.08]
Gender identity (other vs. female)	−0.90	0.31	0.005 **	[−1.52, −0.28]	−0.40	0.28	0.148	[−0.95, 0.14]	−0.41	0.28	0.137	[−0.96, 0.13]
Group status (minority vs. dominant)	−0.19	0.13	0.161	[−0.45, 0.08]	−0.12	0.12	0.303	[−0.35, 0.11]	−0.19	0.12	0.116	[−0.43, 0.05]
History of past twinning (yes vs. no)	0.02	0.14	0.897	[−0.26, 0.30]	−0.06	0.12	0.639	[−0.30, 0.18]	−0.06	0.12	0.613	[−0.30, 0.18]
Extraversion (BFI-E)					0.09	0.04	0.028 *	[0.01, 0.18]	0.10	0.04	0.016 *	[0.02, 0.19]
Openness (BFI-O)					0.01	0.05	0.867	[−0.09, 0.10]	−0.00	0.05	0.928	[−0.10, 0.09]
Appetite for cross-cultural engagement-Attitude subscale (ABOS-A)					0.28	0.07	<0.001 ***	[0.14, 0.42]	0.27	0.07	<0.001 ***	[0.13, 0.41]
Appetite for cross-cultural engagement-Participation subscale (ABOS-P)					0.13	0.08	0.102	[−0.03, 0.29]	0.15	0.08	0.068	[−0.01, 0.30]
Ethnocentrism (RES)					0.00	0.01	0.92	[−0.01, 0.01]	−0.00	0.01	0.657	[−0.02, 0.01]
Intercultural communication anxiety (PRICA)					−0.47	0.10	<0.001 ***	[−0.68, −0.26]	−0.49	0.10	<0.001 ***	[−0.69, −0.28]
Outgroup prejudice (OutPrej)					0.00	0.00	0.232	[−0.00, 0.01]	0.00	0.00	0.590	[−0.00, 0.01]
Cultural congruity in Quebéc (CCS)									−0.12	0.06	0.039 *	[−0.24, −0.01]

Note: * *p* < 0.05; ** *p* < 0.01; *** *p* < 0.001.

### 3.4. Contact Opt-In (BehProx) as Dependent Variable

Table 6 shows the unstandardized results of multiple regressions predicting behavioral proxy scores. Model 1 with sociodemographic covariates was statistically significant, McFadden pseudo-R^2^ = 0.07, *F*(5, 206) = 3.55, *p* = 0.004. Age and history of past twinnings were significantly associated with contact opt-in scores, such that older participants and participants who had never taken part in intercultural twinnings were more likely to provide their email address to take part in future intercultural twinnings. In Model 2, the introduction of correlates documented in previous studies was a significant improvement, R^2^ = 0.17, ΔR^2^ = 0.10, *F*(7, 197.9) = 2.74, *p* = 0.006. Greater ethnocentrism, indexed by RES scores, was associated with lower behavioral intent. For a one-point increase in RES scores, there was a 4% decrease in the likelihood of agreeing to be contacted by email. The ABOS-A coefficient was statistically significant, such that for a one-point increase in appetite for cross-cultural engagement (attitudes scale), there was an 89% increase in the likelihood of agreeing to be contacted by email. In Model 3, the improvement was significant, R^2^ = 0.19, ΔR^2^ = 0.02, *F*(1, 179.9) = 4.82, *p* = 0.03. Perceptions of lower cultural fit in Québec society, as indexed by CCS scores, were associated with greater behavioral intent. Specifically, a one-point increase in CCS scores was related to a 37% decrease in the likelihood of agreeing to be contacted by email for future intercultural twinnings, providing support for our hypothesis.

Results were robust to excluding participants with very short completion times (less than 10 min) and those who completed the survey late in the evening (after 21:00), suggesting that the observed effects are not driven by low engagement or survey fatigue.

### 3.5. Exploratory Analyses: Moderation by Group Status

In a supplementary exploratory step, we tested interactions between cultural congruity scores and group status for each dependent variable. None of these interactions were statistically significant: *B* = −0.13, SE = 0.21, *p* = 0.28 for contact willingness; *B* = −0.11, SE = 0.11, *p* = 0.31 for intrinsic motivation; and *B* = −0.15, SE = 0.34, *p* = 0.66 for behavioral intent. This indicates that the negative association between perceived cultural fit in Québec and outcome measures is independent of dominant vs. minority group status.

### 3.6. Supplementary Analyses: Diagnosing Suppression Effects

Bivariate correlations show that cultural congruity is negatively associated with avoidance-related correlates (ranging from −0.36 to −0.44), but has non-significant zero-order correlations with motivational dependent variables. However, a significant negative association between cultural congruity and motivational variables emerges in multiple regressions. This pattern is consistent with a suppression effect.

We further diagnosed this suppression effect using commonality analyses to decompose effects in unique and shared variance. These results, reported in Supplementary Materials, show that cultural congruity exhibited a non-trivial unique contribution to each outcome (ranging from approximately 6% to 48% of explained variance). Further, shared variance components involving cultural congruity and avoidance-related correlates were consistently negative across models, indicating that variance in cultural congruity shared with avoidance dispositions suppresses its association with motivational variables at the bivariate level. They were also robust to the inclusion of ABOS (attenuated but same pattern).

**Table 6:** Multiple logistic regressions predicting contact opt-in to participate in intercultural twinnings (BehProx).

	Model 1					Model 2					Model 3				
Predictor	*b*	SE	*p*	95% CI	OR	*b*	SE	*p*	95% CI	OR	*b*	SE	*p*	95% CI	OR
Intercept	−1.34	0.65	0.040 *	[−2.62, −0.06]	0.26	−3.18	1.62	0.052	[−6.39, 0.03]	0.04	0.11	2.17	0.959	[−4.16, 4.38]	1.12
Age	0.09	0.03	<0.001 ***	[0.04, 0.14]	1.10	0.11	0.03	<0.001 ***	[0.05, 0.17]	1.12	0.10	0.03	<0.001 ***	[0.05, 0.16]	1.11
Gender identity (male vs. female)	−0.33	0.35	0.345	[−1.02, 0.36]	0.72	0.09	0.41	0.832	[−0.71, 0.89]	1.09	−0.02	0.41	0.961	[−0.84, 0.80]	0.98
Gender identity (other vs. female)	−0.35	0.75	0.643	[−1.83, 1.13]	0.71	0.31	0.84	0.710	[−1.35, 1.97]	1.37	0.27	0.86	0.756	[−1.43, 1.97]	1.31
Group status (minority vs. dominant)	−0.47	0.32	0.144	[−1.09, 0.16]	0.63	−0.21	0.35	0.555	[−0.90, 0.48]	0.81	−0.51	0.39	0.184	[−1.27, 0.25]	0.6
History of past twinning (yes vs. no)	−0.89	0.34	0.010 *	[−1.56, −0.22]	0.41	−1.08	0.38	0.005 **	[−1.83, −0.33]	0.34	−1.12	0.39	0.004 **	[−1.88, −0.36]	0.33
Extraversion (BFI-E)						0.19	0.13	0.157	[−0.07, 0.46]	1.21	0.23	0.14	0.098	[−0.04, 0.51]	1.26
Openness (BFI-O)						−0.06	0.15	0.664	[−0.35, 0.22]	0.94	−0.11	0.15	0.47	[−0.41, 0.19]	0.9
Appetite for cross-cultural engagement-Attitude subscale (ABOS-A)						0.64	0.22	0.005 **	[0.20, 1.08]	1.89	0.65	0.23	0.005 **	[0.20, 1.10]	1.91
Appetite for cross-cultural engagement-Participation subscale (ABOS-P)						−0.03	0.24	0.915	[−0.50, 0.45]	0.97	0.03	0.25	0.898	[−0.46, 0.52]	1.03
Ethnocentrism (RES)						−0.04	0.02	0.039 *	[−0.08, −0.00]	0.96	−0.06	0.02	0.010 *	[−0.10, −0.01]	0.94
Intercultural communication anxiety (PRICA)						−0.18	0.31	0.555	[−0.79, 0.42]	0.83	−0.23	0.33	0.477	[−0.87, 0.41]	0.79
Outgroup prejudice (OutPrej)						−0.00	0.01	0.995	[−0.02, 0.02]	1.00	−0.01	0.01	0.567	[−0.02, 0.01]	0.99
Cultural congruity in Québec (CCS)											−0.46	0.21	0.029 *	[−0.86, −0.05]	0.63

Note: * *p* < 0.05; ** *p* < 0.01; *** *p* < 0.001.

## 4. Discussion

This study tested correlates of motivation for intercultural twinnings—an optimal form of contact highly relevant in our local university context—distinguishing between contact willingness, intrinsic motivation, and contact opt-in as a behavioral proxy. In particular, we introduced and tested the lack of cultural congruity as a novel potential correlate inspired by push–pull theories of migration. The results supported our hypothesis. We found that lower cultural congruity in Québec was associated with significantly more contact willingness, intrinsic motivation, and contact opt-in to participate in future intercultural twinnings, with modest effect sizes.

Outside of the self-expansion proxy, cultural congruity is the only correlate that was consistently associated with all three facets of motivation. The results suggested a suppression effect. Indeed, although zero-order correlations between cultural congruity and the motivational outcomes were weak or non-significant, significant associations emerged in multivariate models once avoidance-related covariates were included. Commonality analyses further clarified this pattern by decomposing the explained variance into unique and shared components. Across outcomes, cultural congruity accounted for a meaningful share of unique variance, despite its weak bivariate associations. Importantly, shared variance components involving cultural congruity and avoidance-related correlates (ethnocentrism, intergroup anxiety, and outgroup prejudice) were consistently negative, a pattern characteristic of suppression. This indicates that variance in cultural congruity associated with avoidance dispositions masks its positive association with motivation in zero-order analyses, but is revealed once this variance is accounted for.

The strength of this pattern varied across outcomes, being most pronounced for willingness to engage and present, though more modest, for intrinsic motivation and the behavioral proxy. Taken together, these findings suggest that cultural congruity does not operate as a direct motivational “push,” but rather as part of a conditional structure in which its association with motivation depends on the interplay with avoidance-related dispositions. More broadly, they point to a meaningful structure in which motivational openness and avoidance processes are intertwined, and highlight the need for future research to more precisely map the interrelations among these correlates to refine theoretical models of contact motivation.

These results suggest that not fitting in well in society—a hitherto unexamined construct—may be a relevant correlate of one’s motivation to take part in intergroup contact, once avoidance dispositions have been taken into account. The mechanisms underlying this association are unknown. They may also differ, depending on one’s dominant-minority status. For example, for minority members, the role of cultural incongruity may be explained by perceived ethnocultural discrimination. Indeed, minority participants felt they fit significantly less well in Québec than dominant-status members, which could be explained by the unfortunate prevalence of discrimination against ethnocultural minorities. In contrast, for members of the dominant group, a feeling that the mainstream does not represent one’s values may explain the association. This mechanism would actually be consistent with the current sociopolitical situation in Québec. The fairly conservative provincial government was elected largely through the vote of citizens outside of Québec’s large cities, where this study was conducted. Thus, participants in this study might not feel well represented by Québec’s government and official image. From a self-determination motivational perspective (Ryan & Deci, 2018), individuals are likely to have greater intrinsic motivation toward contact situations that fulfill basic psychological needs for relatedness (Böttcher & Friehs, 2022). Lower cultural congruity with the mainstream may reflect lower relatedness need satisfaction in that social space and the hope that this need could be satisfied in alternative social contexts, thus driving intrinsic motivation for intercultural twinnings. It would be important for future research to probe such potential mechanisms to better understand how lack of cultural congruity is linked to intergroup contact motivation.

In a related vein, future research could also probe additional potential correlates inspired by push–pull theories of migration. Cultural congruity was one such correlate, but others such as social class or social capital might also be relevant. Further, cultural congruity was associated with motivation to partake in a very specific type of intergroup contact. Intercultural twinnings have fairly little volitional control: individuals typically do not choose with whom they will interact and interactions follow specific guidelines (e.g., conversation topic, length, location). Some research suggests that mandated contact could be more beneficial for intergroup relations (Hodson, 2008; Pettigrew & Tropp, 2006) and does not negatively impact relationship quality (Albuja et al., 2024) compared to freely chosen contact. Whether this association between cultural congruity and contact motivation would generalize to other types of contact is an open question that future research should consider.

Our analyses included a range of traditional meso- and micro-level correlates of contact motivation. The only correlate that was consistently associated with all three facets of motivation is the attitudes facet of appetite for cross-cultural engagement, which served as a proxy for self-expansion. The other ones differed depending on the motivation facet tested. Specifically, lower intergroup anxiety and greater extraversion were related to greater intrinsic motivation and greater ethnocentrism was associated with greater willingness to be contacted, which served as a proxy for behavioral intent. As noted earlier, motivation conceptualizations are manifold. They are also typically not compared head-to-head in a single study. Yet, our results suggest that how we conceptualize motivation does matter and that different facets of motivation may be associated with different sets of correlates. It would be important for future research to map out these differential associations to better understand the various motivation profiles that exist.

To the best of our knowledge, little to no research has investigated correlates of intrinsic motivation for intercultural contact, conceptualized here as a desire to take part in intercultural twinnings because such an activity would be satisfying in and of itself. The significant coefficient for appetite for cross-cultural engagement (attitudes subscale) was unsurprising, given that intercultural twinnings may be a way to satisfy this appetite. Indeed, Böttcher and Friehs (2022) note important conceptual ties between self-expansion and intrinsic motivation. Similarly, the significant negative coefficient for intercultural anxiety could easily be explained by the fact that the anxiety a person experiences could prevent them from genuinely enjoying contact experiences. They may still be willing to engage in intercultural twinnings, but for other reasons. In that vein, it would be interesting for future research to investigate correlates of other types of motivation, such as extrinsic motivation. It is likely that a different motivational profile would emerge.

It would also be useful to think more systematically about the intrinsic motivational aspects of twinnings—and perhaps contact interventions more generally. Self-determination posits that intrinsic motivation is tied to satisfaction of basic competence, autonomy and relatedness needs (Ryan & Deci, 2018). Thus, it may be useful to investigate and highlight how contact interventions could satisfy these needs. For example, addressing the relatedness need could be done by emphasizing similarities of the interaction partners, a technique that has been shown to improve cross-group relationships (e.g., Turner & Cameron, 2016). This understanding could help with a motivational challenge Landry and Halperin (2025) have identified in intergroup contact interventions (and intergroup interventions more broadly) such as intercultural twinnings. They point out that people often lack motivation to engage in such promising interventions, which generally require time and effort. To show that these interventions are successful for intergroup relations, researchers have traditionally removed these motivational barriers by providing incentives such as financial compensation or course credit, which limits their real-world applicability. Landry and Halperin (2025) suggest that satisfying alternative psychological motivations could help address this motivational challenge without resorting to these traditional incentives. More broadly, this calls for exploring how self-determination theory concepts could fruitfully intersect with the intergroup contact literature, Böttcher and Friehs (2022) have argued recently.

### Limitations

A key limitation pertains to the cross-sectional design of our study, preventing us from drawing any conclusion regarding the directionality of effects. Future research should probe the role of cultural congruity (and other correlates) longitudinally. Also, our choice of a student sample allowed us to specify an ecologically valid context for intergroup contact, but it also limits the generalizability of our results. Students are on average younger than the general population and universities are typically left-leaning, or more progressive, environments (van de Werfhorst, 2020) where cultural diversity and intercultural contact are seen as desirable. Both features could affect intergroup contact motivational profiles. In terms of motivation, probing intrinsic motivation and overall motivation level was a strength of this study, but limiting our investigation of motivation to its intrinsic variety is a limitation. It would have been relevant to also include, e.g., extrinsic motivation. In future research, it would be important to study the construct of motivation for intergroup contact more exhaustively, drawing on the extensive motivation literature.

In addition, the behavioral proxy used in this study—agreement to be contacted for future participation—should be interpreted as a low-cost initial commitment (contact opt-in) rather than enacted behavior. Such decisions may be influenced not only by intergroup motivation, but also by factors such as privacy concerns or perceived time availability. As a result, this measure likely contains additional sources of variability unrelated to motivation and should be interpreted cautiously. Future research should examine stronger behavioral indicators, such as actual participation in intercultural activities, to better capture enacted engagement.

The operationalization of group status also warrants caution. The “minority” category aggregates participants with diverse ethnocultural, migratory, and linguistic trajectories, as illustrated in Table 2. While this approach captures a broad structural distinction relative to the dominant group in Québec, it may obscure meaningful within-group differences. Although exploratory analyses indicated that the associations observed in the present study did not differ by group status, future research would benefit from more fine-grained distinctions between subgroup experiences.

A related limitation concerns the measurement of outgroup prejudice. Because the target of this measure varies as a function of participants’ group membership (i.e., dominant vs. minority), it does not capture attitudes toward a single, common target across the sample. As a result, the construct may not be strictly comparable across groups, and differences in its meaning and social implications may complicate interpretation. In the present analyses, this variable is therefore best understood as indexing a within-person disposition toward the salient outgroup rather than a uniform construct. Despite this limitation, the variable functioned as expected as an avoidance-related correlate in the models, suggesting that it captures a meaningful dimension of intergroup orientation, albeit one that should be interpreted with caution.

Further, we probed motivation for generic intercultural twinnings. It is, however, possible for motivation to be more or less keen depending on the twin’s origin. For example, some participants might be more interested in twinning with a European immigrant than with a Maghrebi one—a population that faces significant discrimination in Québec (Valiante, 2017). Dixon et al. (2020) urge us to take into account the “complex relationality of intergroup processes” that are profoundly shaped by historical and sociopolitical forces. This is an area where our study could have been more focused. Finally, we measured cultural misfit with the Québec society, assuming that this is a relevant “ingroup” of sorts for all participants. This may or may not be the case. A better approach in future research might be to first determine participants’ ethnocultural ingroup, including the intersectionality of identity, and then probe the extent to which they fit in in that group.

## 5. Conclusions

Decades of social psychological research have established that intergroup contact is an effective way to reduce intergroup bias and foster harmonious intergroup relations (Pettigrew & Tropp, 2006). However, daily intergroup interactions are typically stressful and scarce (Doucerain et al., 2023) for some. A critical question then becomes how to bring the horse to the intergroup contact water (Pettigrew et al., 2011, p. 278)—also taking into account how power relations affect said water. Better understanding what motivates people to seek or engage in intergroup contact is essential to answer this question. This study took a step in that direction by considering three facets of motivation for an ecologically valid form of intergroup contact, probing a range of traditional correlates as well as cultural congruity, a novel correlate inspired by push–pull theories of migration. A sense of not fitting in well in society emerged as a consistent correlate of contact motivation through a suppression effect (once avoidance dispositions were taken into account).

Better understanding the profile of motivated individuals is helpful because it could help capitalize on their characteristics to leverage contact and direct more concentrated efforts towards those who do not fit this profile. In an age of prevalent intergroup tensions, doing so is essential to help foster harmonious societies. Working in and through education is central to not only understanding lived realities across groups and identities but, importantly, how to create the conditions and dynamics for intercultural living and societies, which is at the base of the intercultural twinning project.

## Figures and Tables

**Table 1:** Summary of documented contact motivation drivers.

Level	Driver	Type of Driver
Macro	Cultural norms (e.g., ethnic tolerance norms) Institutional characteristics	---
Meso	Group status History	---
Micro	Prejudice Social dominance orientation Right-wing authoritarianism Intergroup anxiety	Drive to avoid
Micro	Openness Extraversion Professional advancement goals Group-image concerns Self-expansion	Drive to seek

**Table 2:** Detailed sample composition according to group status.

Group Status	First Language	Racial Background	Nativity	Frequency
Minority	French	Person of Color	Born in Canada	24
	Other language	Person of Color	Born in Canada	4
	Other language	White	Born in Canada	4
	French	Person of Color	Immigrant	20
	Other language	Person of Color	Immigrant	31
	French	White	Immigrant	10
	Other language	White	Immigrant	7
Dominant	French	White	Born in Canada	111

**Table 3:** Descriptive statistics and correlations among numerical variables.

Variables	*M* (SD) Dom	*M* (SD) Min	*M* (SD)	2	3	4	5	6	7	8	9	10	11
1. ContWill	5.05 (1.74)	4.95 (1.79)	5 (1.76)	0.68 ***	0.63 ***	0.11	0.17 *	0.37 ***	0.21 **	−0.16 *	−0.21 **	−0.14	−0.10
2. IntMot	5.1 (0.94)	5 (0.99)	5.05 (0.96)		0.47 ***	0.24 ***	0.19 **	0.31 ***	0.34 ***	−0.24 ***	−0.47 ***	−0.18 *	0.05
3. BehProx	0.65 (0.48)	0.61 (0.49)	0.64 (0.48)			0.08	0.06	0.20 **	0.10	−0.28 ***	−0.22 **	−0.13	0.00
4. BFI-E	4.12 (1.46)	4.02 (1.26)	4.07 (1.37)				0.18 *	0.09	0.21 **	0.01	−0.21 **	−0.10	0.13
5. BFI-O	5.04 (1.3)	4.92 (1.37)	4.98 (1.33)					0.27 ***	0.41 ***	−0.07	−0.11	−0.09	0.00
6. ABOS-A	3.76 (0.85)	3.55 (0.9)	3.66 (0.88)						0.28 ***	−0.13	−0.21 **	−0.32 ***	0.15 *
7. ABOS-P	3.41 (0.87)	3.52 (0.82)	3.46 (0.85)							0.02	−0.29 ***	−0.20 **	0.07
8. RES	24.31 (9.65)	28.82 (11.31)	26.42 (10.67)								0.53 ***	0.30 ***	−0.44 ***
9. PRICA	2.04 (0.64)	2.14 (0.76)	2.08 (0.7)									0.36 ***	−0.36 ***
10. OutPrej	22.27 (17.25)	31.43 (24.76)	26.47 (21.46)										−0.44 ***
11. CCS	5.76 (1.12)	4.86 (1.09)	5.36 (1.19)										

Note. The Dom column provides statistics for participants in the Québec dominant group and the Min column for those in the Québec minority group. * *p* < 0.05; ** *p* < 0.01; *** *p* < 0.001.

## Data Availability

The data that support the findings of this study, along with the analysis script and the study materials, are openly available in the Open Science Framework at https://osf.io/yux3t/overview?view_only=eb69c07965ee426ba09f5ecc84dd42a5, accessed on 23 May 2026.

## Supplementary Materials

The following supporting information can be downloaded at: https://www.mdpi.com/article/10.3390/bs16060921/s1, Table S1. Model fit indices for CFA on CCS, Figure S1. Factor loadings of CFA on CCS, Table S2. Invariance across minority vs. dominant group status, Table S3. Splitting CCS: ContWill as dependent variable, Table S4. Splitting CCS: IntMot as dependent variable, Table S5. Splitting CCS: BehProx as dependent variable, Table S6. ABOS items + ContWill items, EFA factor loadings, Table S7. ABOS items + IntMot items, EFA factor loadings, Table S8. Commonalities-ContWill as dependent variable, Table S9. Commonalities-IntMot as dependent variable, Table S10. Commonalities-BehProx as dependent variable, Table S11. DFBETAs for cultural congruity, Table S12. Variance inflation factors for ContWill, Figure S2. Residuals vs. Fitted values for ContWill, Table S13. Variance inflation factors for IntMot, Figure S3. Residuals vs. Fitted values for IntMot, Table S14. Variance inflation factors for BehProx.
