# Supplementary material for: Fitting in or Not Fitting in: Cultural Congruity as a Correlate of Motivation for Intergroup Contact
Source: Behav Sci (Basel). 2026 Jun 4;16(6):921. doi: 10.3390/bs16060921 (PMC13295900; doi:10.3390/bs16060921)
Supplement: Supplementary file 1 [file behavsci-16-00921-s001.zip › behavsci-4074797-supplementary.pdf]

SUPPLEMENTARY MATERIALS

Table of Contents

I. Brief psychometric evaluation of CCS 2
Table S1. Model fit indices for CFA on CCS 2
Figure S1. Factor loadings of CFA on CCS 2
CCS items, colored to reflect factor loading 2
Table S2. Invariance across minority vs dominant group status 3
II. Exploratory regressions splitting CCS into two facets 3
1. Table S3. Splitting CCS: ContWill as dependent variable 3
2. Table S4. Splitting CCS: IntMot as dependent variable 3
3. Table S5. Splitting CCS: BehProx as dependent variable 4
III. Factor analysis of ABOS + motivational dependent variables 4
1. Table S6. ABOS items + ContWill items, EFA factor loadings 4
2. Table S7. ABOS items + IntMot items, EFA factor loadings 5
IV. Commonalities analysis to diagnose suppression effect 5
1. Table S8. Commonalities - ContWill as dependent variable 5
2. Table S9. Commonalities - IntMot as dependent variable 6
3. Table S10. Commonalities - BehProx as dependent variable 6
V. Sensitivity analyses: role of influential observations for CCS effect 7
Table S11. DFBETAs for cultural congruity 7
VI. Verification of linear model assumptions 7
1. ContWill as dependent variable 7
Table S12. Variance inflation factors for ContWill 7
Figure S2. Residuals vs. Fitted values for ContWill 8
2. IntMot as dependent variable 8
Table S13. Variance inflation factors for IntMot 8
Figure S3. Residuals vs. Fitted values for IntMot 9
3. BehProx as dependent variable 9
Table S14. Variance inflation factors for BehProx 9

I. Brief psychometric evaluation of CCS

Confirmatory Factor Analysis of CCS items (7 indicators), 2-factor model

Table S1. Model fit indices for CFA on CCS

	χ^2	df	χ^2/df	p	CFI	TLI	RMSEA [90% CI]	SRMR	AIC	BIC
Model	22.04	13	1.70	.055	.98	.98	.06 [.00, .10]	.03	4,536.76	4,585.07
**Common guidelines[a]**	—	—	< 2 or 3	> .05	≥ .95	≥ .95	< .05 [.00, .08]	≤ .08	Smaller	Smaller

[a]Based on Schreiber (2017), Table 3.

Figure S1. Factor loadings of CFA on CCS

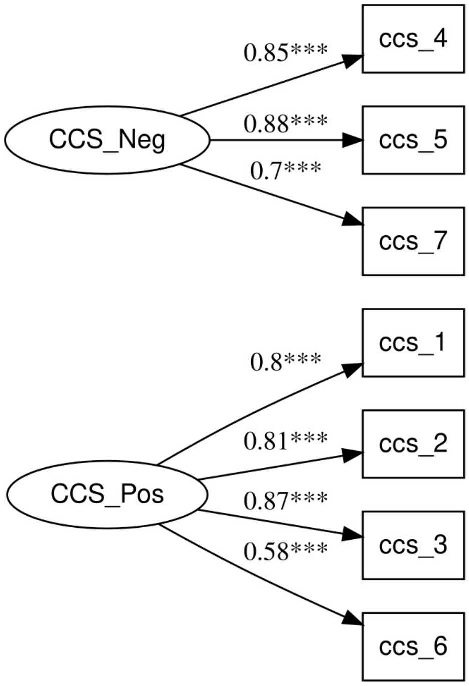

CCS items, colored to reflect factor loading

1. Je sens que j'ai ma place au Québec.
2. Lorsque j'interagis avec des Québécois·es, je sens que nous sommes sur la même longueur d'onde.
3. Je me sens à l'aise lorsque je travaille avec des Québécois·es.
4. Lorsque j'interagis avec des Québécois·es, je me sens de trop.
5.Je sens le besoin de changer qui je suis afin d'avoir ma place au sein des Québecois·es.
6. Lorsque j'interagis avec des Québécois·es je sais intuitivement comment me comporter ou comment réagir.
7. Je ne comprends pas vraiment les références culturelles qu'utilisent les Québécois·es.

Blue items: positively worded

Green items: negatively worded

Table S2. Invariance across minority vs dominant group status

Model	χ^2	*df*	*p*	CFI	TLI	RMSEA	SRMR	ΔCFI	ΔRMSEA
Configural	31.62	26	.206	.991	.985	.048*	.047*		
Metric	39.46	31	.142	.986	.981	.054	.059	-.005***	.006**

II. Exploratory regressions splitting CCS into two facets

1. Table S3. Splitting CCS: ContWill as dependent variable

Term	*b*	SE	*p*	95% CI
Intercept	2.87	1.38	.040*	[0.13, 5.60]
Age	0.08	0.02	< .001***	[0.04, 0.11]
Gender identity (male vs. female)	-0.43	0.26	.092	[-0.94, 0.07]
Gender identity (other vs. female)	0.14	0.54	.795	[-0.92, 1.20]
Group status (minority vs. dominant)	-0.39	0.24	.109	[-0.86, 0.09]
History of past twinning (yes vs. no)	-0.33	0.24	.169	[-0.80, 0.14]
Extraversion (BFI-E)	0.12	0.08	.167	[-0.05, 0.28]
Openness (BFI-O)	-0.02	0.09	.830	[-0.20, 0.16]
Appetite for cross-cultural engagement - Attitude subscale (ABOS-A)	0.79	0.14	< .001***	[0.52, 1.06]
Appetite for cross-cultural engagement - Participation subscale (ABOS-P)	0.07	0.16	.677	[-0.24, 0.37]
Ethnocentrism (RES)	-0.01	0.01	.364	[-0.04, 0.01]
Intercultural communication anxiety (PRICA)	-0.29	0.21	.180	[-0.71, 0.13]
Outgroup prejudice (OutPrej)	-0.00	0.01	.696	[-0.01, 0.01]
Cultural congruity in Quebec - Positive items (CCS-P)	-0.25	0.10	.015*	[-0.44, -0.05]
Cultural congruity in Quebec - Negative items (CCS-N)	-0.14	0.08	.089	[-0.31, 0.02]

2. Table S4. Splitting CCS: IntMot as dependent variable

Term	b	SE	p	95% CI
Intercept	4.19	0.71	< .001***	[2.78, 5.60]
Age	0.03	0.01	< .001***	[0.01, 0.05]
Gender identity (male vs. female)	-0.17	0.13	.185	[-0.44, 0.08]
Gender identity (other vs. female)	-0.41	0.27	.138	[-0.95, 0.13]
Group status (minority vs. dominant)	-0.20	0.12	.105	[-0.44, 0.04]
History of past twinning (yes vs. no)	-0.05	0.12	.692	[-0.29, 0.19]
Extraversion (BFI-E)	0.10	0.04	.014*	[0.02, 0.19]
Openness (BFI-O)	-0.00	0.05	.931	[-0.10, 0.09]
Appetite for cross-cultural engagement - Attitude subscale (ABOS-A)	0.27	0.07	< .001***	[0.13, 0.41]
Appetite for cross-cultural engagement - Participation subscale (ABOS-P)	0.14	0.08	.074	[-0.01, 0.30]
Ethnocentrism (RES)	-0.00	0.01	.668	[-0.02, 0.01]

Term	b	SE	p	95% CI
Intercultural communication anxiety (PRICA)	-0.48	0.11	< .001***	[-0.69, -0.27]
Outgroup prejudice (OutPrej)	0.00	0.00	.598	[-0.00, 0.01]
Cultural congruity in Quebec - Positive items (CCS-P)	-0.08	0.05	.129	[-0.17, 0.02]
Cultural congruity in Quebec - Negative items (CCS-N)	-0.05	0.04	.245	[-0.13, 0.03]

3. Table S5. Splitting CCS: BehProx as dependent variable

Term	b	SE	p	or	95% CI
Intercept	-1.15	2.27	.613	0.32	[-5.64, 3.34]
Age	0.11	0.03	< .001***	1.12	[0.05, 0.17]
Gender identity (male vs. female)	-0.05	0.43	.909	0.95	[-0.90, 0.80]
Gender identity (other vs. female)	0.13	0.91	.889	1.14	[-1.67, 1.93]
Group status (minority vs. dominant)	-0.65	0.40	.107	0.52	[-1.44, 0.14]
History of past twinning (yes vs. no)	-1.06	0.39	.007**	0.35	[-1.83, -0.29]
Extraversion (BFI-E)	0.26	0.14	.064	1.30	[-0.02, 0.54]
Openness (BFI-O)	-0.08	0.15	.609	0.92	[-0.38, 0.22]
Appetite for cross-cultural engagement - Attitude subscale (ABOS-A)	0.79	0.24	.001**	2.20	[0.32, 1.26]
Appetite for cross-cultural engagement - Participation subscale (ABOS-P)	0.12	0.26	.638	1.13	[-0.39, 0.64]
Ethnocentrism (RES)	-0.06	0.02	.018*	0.95	[-0.10, -0.01]
Intercultural communication anxiety (PRICA)	-0.08	0.35	.823	0.92	[-0.76, 0.60]
Outgroup prejudice (OutPrej)	-0.01	0.01	.288	0.99	[-0.03, 0.01]
Cultural congruity in Quebec - Positive items (CCS-P)	-0.57	0.18	.002**	0.56	[-0.94, -0.20]
Cultural congruity in Quebec - Negative items (CCS-N)	0.06	0.13	.641	1.07	[-0.20, 0.33]

III. Factor analysis of ABOS + motivational dependent variables

Principal axis factoring with promax rotation

1. Table S6. ABOS items + ContWill items, EFA factor loadings

	Factor 1	Factor 2	Factor 3
ABOS_1	0.06	-0.02	**0.77**
ABOS_2	-0.23	-0.10	**0.81**
ABOS_3	0.06	-0.10	**0.76**
ABOS_4	0.11	0.08	**0.52**
ABOS_5	*0.27*	0.12	**0.22**
ABOS_12	0.01	**0.68**	-0.20
ABOS_13	-0.01	**0.61**	-0.08
ABOS_14	-0.03	**0.68**	0.02
ABOS_15	-0.03	**0.42**	0.04

ABOS_16	-0.08	**0.41**	0.20
ABOS_17	0.17	**0.47**	-0.02
ABOS_18	-0.02	**0.71**	-0.03
ABOS_19	-0.07	**0.56**	0.02
ContWill_1	**0.97**	-0.06	-0.04
ContWill_2	**0.97**	-0.05	-0.03
ContWill_3	**0.99**	-0.08	-0.06

2. Table S7. ABOS items + IntMot items, EFA factor loadings

	Factor 1	Factor 2	Factor 3
ABOS_1	-0.05	0.03	**0.80**
ABOS_2	-0.10	-0.09	**0.70**
ABOS_3	-0.08	-0.04	**0.82**
ABOS_4	0.27	0.00	**0.52**
ABOS_5	0.10	0.13	**0.29**
ABOS_12	0.04	**0.62**	-0.16
ABOS_13	0.00	**0.54**	-0.03
ABOS_14	-0.13	**0.66**	0.08
ABOS_15	0.04	**0.40**	0.01
ABOS_16	0.09	**0.33**	0.16
ABOS_17	0.11	**0.50**	-0.00
ABOS_18	-0.20	**0.83**	0.00
ABOS_19	-0.15	**0.64**	0.01
IntMot_1	**0.86**	-0.09	0.07
IntMot_2	**0.37**	0.15	0.00
IntMot_3	**0.39**	-0.17	0.04
IntMot_4	**0.50**	0.20	-0.06
IntMot_5	**0.73**	-0.05	0.12
IntMot_6	**0.29**	*0.21*	-0.07
IntMot_7	**0.27**	-0.03	-0.10
IntMot_8	**0.81**	-0.02	0.08

IV. Commonalities analysis to diagnose suppression effect

1. Table S8. Commonalities - ContWill as dependent variable

	Commonality coefficient	Percent of total
Unique to RES	0.01	10.63
Unique to PRICA	0.03	26.12

Unique to OutPrej	0.02	13.07
Unique to CCS	0.06	48.46
Common to RES, and PRICA	0.03	25.53
Common to RES, and OutPrej	0.00	0.05
Common to PRICA, and OutPrej	0.01	10.80
Common to RES, and CCS	-0.01	-8.65
Common to PRICA, and CCS	-0.01	-5.39
Common to OutPrej, and CCS	-0.01	-11.24
Common to RES, PRICA, and OutPrej	0.01	7.56
Common to RES, PRICA, and CCS	-0.01	-11.84
Common to RES, OutPrej, and CCS	0.00	0.32
Common to PRICA, OutPrej, and CCS	-0.01	-5.83
Common to RES, PRICA, OutPrej, and CCS	0.00	0.40
Total	0.12	100.00

2. Table S9. Commonalities - IntMot as dependent variable

	Commonality coefficient	Percent of total
Unique to RES	0.00	0.22
Unique to PRICA	0.17	67.38
Unique to OutPrej	0.00	1.02
Unique to CCS	0.02	6.31
Common to RES, and PRICA	0.05	18.39
Common to RES, and OutPrej	-0.00	-0.00
Common to PRICA, and OutPrej	0.02	7.50
Common to RES, and CCS	0.00	0.03
Common to PRICA, and CCS	-0.01	-2.86
Common to OutPrej, and CCS	-0.00	-0.89
Common to RES, PRICA, and OutPrej	0.01	3.32
Common to RES, PRICA, and CCS	-0.01	-3.21
Common to RES, OutPrej, and CCS	-0.00	-0.00
Common to PRICA, OutPrej, and CCS	-0.00	-1.85
Common to RES, PRICA, OutPrej, and CCS	0.01	4.63
Total	0.25	100.00

3. Table S10. Commonalities - BehProx as dependent variable

	Commonality coefficient	Percent of total
Unique to RES	0.05	39.56
Unique to PRICA	0.01	9.76
Unique to OutPrej	0.01	6.53

Unique to CCS	0.03	23.83
Common to RES, and PRICA	0.04	31.65
Common to RES, and OutPrej	0.00	0.11
Common to PRICA, and OutPrej	0.01	4.56
Common to RES, and CCS	-0.02	-13.42
Common to PRICA, and CCS	-0.00	-2.27
Common to OutPrej, and CCS	-0.01	-5.49
Common to RES, PRICA, and OutPrej	0.01	7.48
Common to RES, PRICA, and CCS	-0.01	-4.94
Common to RES, OutPrej, and CCS	0.00	1.24
Common to PRICA, OutPrej, and CCS	-0.00	-2.57
Common to RES, PRICA, OutPrej, and CCS	0.00	3.97
Total	0.12	100.00

V. Sensitivity analyses: role of influential observations for CCS effect

To examine the potential influence of individual observations, we used DFBETAs for cultural congruity. We reran regressions excluding cases exceeding conventional thresholds ($|DFBETA| > 2/\sqrt{n}$).

Table S11. DFBETAs for cultural congruity

Dependent variable	*b*(SE) with all observations (in ms)	*b*(SE) without influential observations
Willingness to participate (ContWill)	-0.40(0.12), $p<.001$	-0.60(0.12), $p<.001$
Intrinsic motivation (IntMot)	-0.12(0.06), $p=.04$	-0.19(0.06), $p=.003$
Behavioral proxy (BehInt)	-0.46(0.21), $p=.03$	-0.12(0.04), $p=.001$

VI. Verification of linear model assumptions

1. ContWill as dependent variable

Table S12. Variance inflation factors for ContWill

Term	VIF
Intercept	
Age	1.35
Gender identity (male vs. female)	1.24
Gender identity (other vs. female)	1.24
Group status (minority vs. dominant)	1.33
History of past twinning (yes vs. no)	1.09
Extraversion (BFI-E)	1.15

Term	VIF
Openness (BFI-O)	1.30
Appetite for cross-cultural engagement - Attitude subscale (ABOS-A)	1.36
Appetite for cross-cultural engagement - Participation subscale (ABOS-P)	1.56
Ethnocentrism (RES)	1.83
Intercultural communication anxiety (PRICA)	1.85
Outgroup prejudice (OutPrej)	1.48
Cultural congruity in Quebec (CCS)	1.75

Figure S2. Residuals vs. Fitted values for ContWill

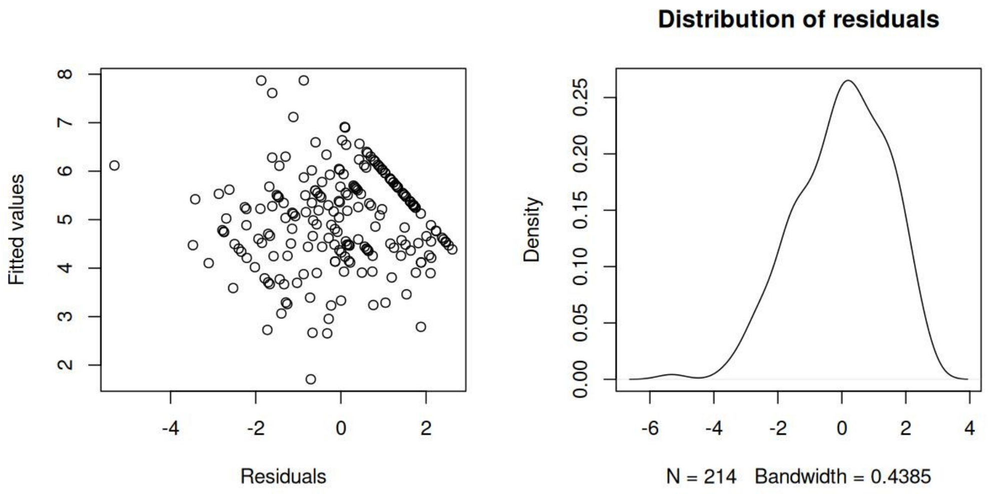

2. IntMot as dependent variable

Table S13. Variance inflation factors for IntMot

Term	VIF
Intercept	
Age	1.35
Gender identity (male vs. female)	1.24
Gender identity (other vs. female)	1.24
Group status (minority vs. dominant)	1.33
History of past twinning (yes vs. no)	1.09
Extraversion (BFI-E)	1.15
Openness (BFI-O)	1.30
Appetite for cross-cultural engagement - Attitude subscale (ABOS-A)	1.36
Appetite for cross-cultural engagement - Participation subscale (ABOS-P)	1.56
Ethnocentrism (RES)	1.83
Intercultural communication anxiety (PRICA)	1.85

Term	VIF
Outgroup prejudice (OutPrej)	1.48
Cultural congruity in Quebec (CCS)	1.75

Figure S3. Residuals vs. Fitted values for IntMot

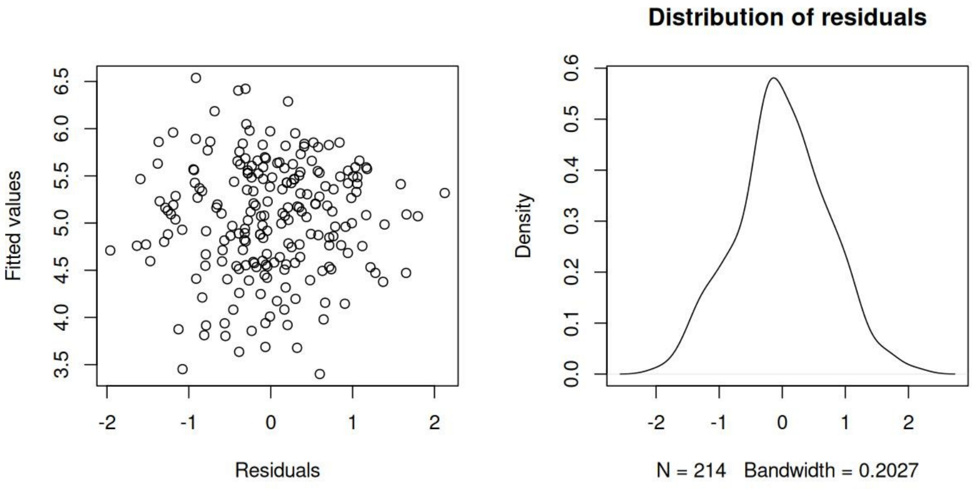

3. BehProx as dependent variable

Table S14. Variance inflation factors for BehProx

Term	VIF
Intercept	
Age	1.42
Gender identity (male vs. female)	1.31
Gender identity (other vs. female)	1.31
Group status (minority vs. dominant)	1.37
History of past twinning (yes vs. no)	1.18
Extraversion (BFI-E)	1.26
Openness (BFI-O)	1.33
Appetite for cross-cultural engagement - Attitude subscale (ABOS-A)	1.42
Appetite for cross-cultural engagement - Participation subscale (ABOS-P)	1.55
Ethnocentrism (RES)	1.94
Intercultural communication anxiety (PRICA)	1.82
Outgroup prejudice (OutPrej)	1.42
Cultural congruity in Quebec (CCS)	2.02
